# Hydrogen Sulfide Signaling in Oxidative Stress and Aging Development

**DOI:** 10.1155/2015/357824

**Published:** 2015-05-17

**Authors:** Guangdong Yang, Steven S. An, Yong Ji, Weihua Zhang, Yanxi Pei

**Affiliations:** ^1^School of Kinesiology, Lakehead University, Thunder Bay, Canada P7B 5E1; ^2^Department of Environmental Health Sciences, Bloomberg School of Public Health, Johns Hopkins University, Baltimore, MD 21218, USA; ^3^Department of Pathophysiology, Nanjing Medical University, Nanjing 210029, China; ^4^Department of Pathophysiology, Harbin Medical University, Harbin 150081, China; ^5^School of Life Science, Shanxi University, Taiyuan 030006, China

Just in the last decade, the knowledge of the physiologic functions of hydrogen sulfide (H_2_S) in medicine and biology has been tremendously accumulated [1–3]. H_2_S has emerged to be one important member of the family of gasotransmitters, together with nitric oxide (NO) and carbon monoxide (CO) [1, 2]. The ubiquitous distribution of H_2_S-producing enzymes and the potent chemical reactivities of H_2_S in biological system make this molecule unique in regulating cellular and organ functions in both the mammalian and plants [1–3]. Pathophysiological abnormalities related to altered H_2_S metabolism and function have been demonstrated in many cases [2, 3]. H_2_S possesses a great therapeutic potential in age-associated diseases by modulation of oxidative stress [4, 5].

This special issue contains review paper and research articles that focus on the topics of H_2_S signaling in oxidative stress and aging development, including discussions on the potency and efficiency of H_2_S in dealing with various diseases. A number of contributions have addressed the protective role of H_2_S in cardiovascular diseases and diabetes. In an original research article, H. Yu et al. demonstrate that H_2_S decreases NADPH oxidase activity and reactive oxidative species (ROS) production, which lead to reduced mean arterial pressure and heart rate in spontaneously hypertensive rats. H_2_S, as an antioxidant, may be a potential target for cardiovascular diseases. A research article by S. Jin and colleagues compares H_2_S generation in ageing diabetic mouse hearts, and they find that H_2_S levels are reduced in the diabetic heart due to the alterations in H_2_S-producing enzymes, which might be related with the pathogenesis of diabetic cardiomyopathy. Y. Zong and colleagues explore the possible effects of endogenous H_2_S on endothelial apoptosis under high-salt stimulation, and their data validate that supplementation of H_2_S donor markedly inhibits vascular endothelial cell oxidative stress and mitochondria-related apoptosis induced by high salt. Q. Wang and colleagues report that H_2_S antagonizes advanced glycation end-products induced-epithelial sodium channel activity by targeting the ROS/PI3K/PTEN pathway in A6 cells. The authors conclude that H_2_S may provide protection against hypertension in diabetic patients. In a review paper by Y. Shen and colleagues, the underlying mechanisms for the cardioprotective effects of H_2_S against myocardial infarction, arrhythmia, hypertrophy, heart failure, and so forth are discussed. Some mechanisms, including antioxidative action, preservation of mitochondrial function, reduction of apoptosis, anti-inflammatory responses, angiogenic actions, regulation of ion channel, and interaction with NO, are mostly responsible for the cardioprotective effect of H_2_S.

Some papers in this special issue describe new insights into the therapeutic potential in fibrosis. In a review paper, S. Zhang and colleagues summarize studies that supplement with exogenous H_2_S mitigates the severity of fibrosis in various experimental animal models. The protective role of H_2_S in the development of fibrosis is primarily attributed to its antioxidation, antiapoptosis, anti-inflammation, proangiogenesis, and inhibition of fibroblasts activities. K. Song and colleagues continue on with this topic that H_2_S protects fibrosis diseases that relate to heart, liver, kidney, and other organs. In a research article, G. Meng and colleagues provide new evidence on the protective role of GYY4137, a slow-releasing H_2_S donor, in myocardial fibrosis by inhibiting oxidative stress, blocking TGF-*β*1/Smad2 signaling pathway, and decreasing in expression of *α*-SMA. Further clinical studies are needed to translate this potential to clinical use.

D. Wu and colleagues highlight the recent findings regarding the role of H_2_S in ischemia-reperfusion (I/R) injury. In their paper, the authors proposed that treatment with H_2_S or its donors in proper dose range and time frame will exhibit more potent therapeutic effects against I/R injury in further preclinical research and clinical application. A review article by W. Zhang and her colleagues addresses the reciprocal interaction between H_2_S and calcium ion channels and transporters through different mechanisms, all of which are essential for the maintenance of intracellular calcium homeostasis by H_2_S. In an original research article, L. Zhang and colleagues explore the role of H_2_S in human gastric neoplasias. Their data point that H_2_S level is lower in noncancerous gastric samples in comparison with human gastric carcinoma mucosa, and the authors further prove that H_2_S induces apoptosis and inhibits cell migration and invasion of gastric cancer cells by regulating apoptosis related proteins. The therapeutic application of H_2_S donors against gastric cancer development can be realized.

In a review article, B. Wu and colleagues discuss the latest research on the interaction of H_2_S with oxygen sensing under hypoxia condition. Emerging evidence has elucidated an important protective role of H_2_S in hypoxia-mediated damage in many mammalian systems. By regulating the functions of hypoxia-inducible factors and the activation of carotid bodies, H_2_S acts as important oxygen/hypoxia sensor.

Not only has it acted as a signalling molecule in mammalian system, but also overwhelming evidence has demonstrated that H_2_S plays important roles in diverse physiological processes in plants. J. Zhu and Y. Pei discuss in a review article the physiological implications of H_2_S in plants. H_2_S modulates various defence responses in plants, including growth and development, abiotic stress, heavy metal toxicity, drought and osmotic stress, hypoxia, senescence, and maturation by interacting with plant hormones, hydrogen peroxide, NO, CO, and other molecules. The same research group also provides evidence that H_2_S alleviates cadmium-induced cell death in Chinese cabbage roots, and they further verify that, by upregulating antioxidant enzyme activities, H_2_S removes excessive ROS and reduces cell oxidative damage induced by cadmium. In one original research article, Y. Zhang and colleagues demonstrate that H_2_S acts as an antioxidant in delaying cell apoptosis and enhancing *α*-amylase secretion regardless of the presence of gibberellic acid in barley aleurone layers. In addition, D.-B. Zhu and colleagues investigate the effects of SO_2_ pretreatment on H_2_S and ROS accumulation in germinating wheat seeds, and their data suggest that SO_2_ could increase endogenous H_2_S accumulation and the antioxidant capability and decrease endogenous aluminum content in wheat grain to alleviate aluminum stress. SO_2_ may be reduced to H_2_S by sulfite reductase, thus contributing to H_2_S production.

The articles presented in this special issue highlight the current advances in the research field of H_2_S in medicine and biology. These articles not only enrich our understanding of how H_2_S regulation of oxidative stress in various disorders occurs but also provide evidence on the therapeutic potential of H_2_S against aging development and other disorders. We hope that readers will find these contributions interesting and informative.
